# Exploring the effect of leader other-oriented perfectionism on radical innovation

**DOI:** 10.3389/fpsyg.2025.1387963

**Published:** 2025-04-14

**Authors:** Le Wang, Yu Wang, Xiu Jin

**Affiliations:** Department of Business Administration, Gachon University, Seongnam, Republic of Korea

**Keywords:** leader’s other-oriented perfectionism, work engagement, radical innovation, conscientiousness, promotion focus

## Abstract

**Introduction:**

Rapid shifts in the global economy have increased the demand for innovation within companies, with radical innovation being a key factor for achieving competitive advantage, organizational success, and sustainable growth. Leadership traits can significantly affect employee behavior and attitudes, which in turn, can influence their work. leader’s others-oriented perfectionism, representing the interaction between a perfectionist leader and their employees, is particularly relevant in the workplace.

**Methods:**

This study constructed a theoretical model to explore the relationship between leadership perfectionism and employee radical innovation and validated it through empirical research.

**Results:**

The findings indicate that there is a positive correlation between leader’s others-oriented perfectionism and employees’ work engagement, which in turn is related to radical innovation. In addition, there is a moderating effect of leader’s conscientiousness between the effects of perfectionism on employee work engagement. Employee promotion focus moderated the mediating role of work engagement in the relationship between leader perfectionism and employee radical innovation.

**Discussion:**

The purpose of this study is to reveal the relationship between leadership perfectionism and employee work engagement and to explore how these findings can help organizations enhance employee breakthrough innovation. The findings will provide specific practical guidance for managers to encourage the combination of leadership perfectionism and accountability to drive employee work engagement and expand employee facilitation focus, ultimately impacting breakthrough innovation.

## 1 Introduction

Today’s world is undergoing profound changes, and the wave of digitization has accelerated global market restructuring, placing higher demands on corporate innovation. Enterprises are faced with both challenges and once-in-a-century opportunities to catch up (Wang et al., 2023). The traditional innovation model can no longer meet the needs of breakthrough development, and it is crucial to enhance the capability of breakthrough innovation (Li et al., 2023). Breakthrough innovation has a significant impact through fundamental improvements beyond existing solutions (Wang et al., 2023). It can reshape the industry landscape and drive business competitiveness and industrial upgrading (Wang et al., 2022). Against the backdrop of heightened global economic uncertainty, breakthrough innovation is not only a source of revolutionary solutions, but also a key to driving economic recovery (Cheng and Wu, 2023). However, the high cost and low success rate of breakthrough innovations discourage many employees (Yang and Wang, 2023). How to incentivize employees to participate in high-risk innovations has become a core issue that companies must address.

With the increasing importance of innovative work in organizational settings, the relationship between perfectionism and innovative behavior has become a focus of research (Park and Shin, 2022). However, much of the existing research has focused on self-oriented perfectionism, ignoring the role of other-oriented perfectionism in the workplace (Stoeber and Rennert, 2008). The present study focuses on leader-other-oriented perfectionism and argues that it is highly relevant to the workplace because it exemplifies leader-employee interactions on perfectionist traits. Perfectionism is a personality trait that manifests itself by striving for perfection, setting extremely high standards, and judging one’s own behavior harshly (Flett and Hewitt, 2002). Although traditional research has tended to focus on the negative effects of perfectionism, recent research has shown that under certain conditions, perfectionism may also produce positive outcomes (Ocampo et al., 2020). Leaders, as perfectionists, tend to set high standards for their employees, emphasizing personal achievement and success, which may motivate employees to go beyond the status quo and strive for excellence (Fowler et al., 2018). This dissatisfaction with the status quo and pursuit of excellence may push employees to break away from existing solutions and experiment with innovative options, which may inspire breakthrough innovation (Xu et al., 2022). Therefore, this study takes leadership perfectionism as a prior variable to explore how leader’s other-oriented perfectionism perfectionism predicts employees’ radical innovation, providing a new theoretical perspective on the role of perfectionism in organizational behavior.

This study focuses on the pathways through which leadership perfectionism enhances employees’ radical innovation and analyzes the mediating mechanisms involved. Work engagement, as a positive affective state, can help employees cope with the high standards demanded by perfectionism and provide them with the energy needed to accomplish difficult tasks (Ocampo et al., 2020). Therefore, this study examined work engagement as a mediating variable. Engaged employees are energized and enthusiastic about their work and perceive it as challenging rather than stressful, thus experiencing positive occupational well-being (Bakker et al., 2014). The increasing demands of modern organizations that expect employees to go beyond the call of duty, take the initiative to innovate, and commit to professional development have increased the need for energetic and engaged employees (Bakker and Schaufeli, 2008). Research has shown that engaged employees are not only able to cope with uncertainty in the innovation process, but also actively contribute to innovative behaviors, which can lead to breakthrough innovations (Gupta et al., 2017). Based on this, this study argues that engaged employees are more likely to adopt proactive behaviors, take work challenges seriously, and ultimately achieve radical innovation when faced with high standards of leader perfectionism. By introducing work engagement as a mediating variable, this study aims to reveal the underlying mechanisms by which leader’s perfectionism promotes employees’ radical innovation.

Personality research increasingly recognizes that one personality trait leads to the behavioral expression of others, for example, the realization that a person’s level of extraversion may amplify or modulate the performance of his or her conscientiousness in a social or work setting considers the interactions between traits, rather than treating traits as separate dimensions (Hofstee et al., 1992; Johnson and Ostendorf, 1993). As a result, predicting and explaining behavior requires a more holistic view of personality (Shoss et al., 2015). According to Tupes and Christal (1992), conscientiousness has been widely studied and is usually characterized by the key dimensions of reliability, organization, persistence and accountability. Leaders with high levels of conscientiousness are typically more resilient and persistent and may be more effective leaders (Judge et al., 2002). Research suggests that leader’s conscientiousness is positively related to other-oriented perfectionism (Hewitt and Flett, 1991) and demonstrates positive effects by encouraging more participatory behaviors (Shoss et al., 2015; Birch et al., 2019). Therefore, this study examines leader’s conscientiousness as a moderating variable to explore how it interacts with leadership perfectionism, which in turn predicts employee engagement. This study provides new perspectives for understanding the impact of leadership personality traits on employee behavior.

This study argues that employees’ radical innovation are realized through a combination of the mediating role of work engagement and the moderating role of psychological characteristics. Therefore, it is necessary to explore how employees’ psychological characteristics modulate this process. Moderating focus theory states that promotion focus is concerned with positive outcomes and favors aggressive strategies (Wu et al., 2021). Promotion focus stimulates positive emotions and makes individuals more optimistic about future expectations, which promotes creativity and innovative performance (Baas et al., 2008). In addition, employees with a promotion focus are more inclined to come up with new ideas and explore new approaches (Zhou et al., 2012). Based on this, this study predicts that promotion focus can interact with employee work engagement to play a positively role on employees’ radical innovation. This study provides a new perspective for understanding the role of employee psychological characteristics in innovative behavior.

Overall, this study builds a unified theoretical framework to integrate the model based on Job demands-resources theory (Demerouti et al., 2001). Leadership perfectionism can be viewed as a challenging demand that motivates employee effort and commitment by setting high standards. Work engagement, on the other hand, is viewed as a positive psychological state, driven by resource adequacy and a reasonable match of demand, which enables employees to think out of the box and realize radical innovations. Conscientiousness as a personality factor provides the resources needed for challenging demands, and finally, promotion focus is a personal resource that helps employees transform challenging demands into positive outcomes.

Given the aforementioned background, this study contributes in the following ways.

First, perfectionism is a significant individual-level variable that affects employee behavior. However, most work to date has focused primarily on clinical, student, and athletic populations, with limited attention on the workplace or specific occupational groups, such as social workers, that may be at higher risk. The nature and effect of perfectionism among social workers remain largely unexplored (Kinman and Grant, 2022). Note that the workplace is a social setting where leaders frequently interact with employees and can assess their performance, making it a crucial context for examining the effect of leaders’ other-oriented perfectionism (Shoss et al., 2015). Consequently, this study focuses on the leader’s perfectionism and investigates its effect on employee behavior.

Second, the focus of research on perfectionism in the workplace has primarily been on individuals’ self-oriented perfectionism (Xu et al., 2022). Kim et al. (2017) concluded that self-oriented perfectionism positively affects employee creativity. However, there has been limited exploration of how a leader’s other-oriented perfectionism affects employees. This study, drawing on Hewitt and Flett's (1991) definition of other-oriented perfectionism, investigates whether a leader’s high perfectionism standards for others can stimulate radical innovation among employees. Current findings suggest that other-oriented perfectionism is not a positive form of perfectionism (Stoeber, 2014). Previous research has found that a leader’s perfectionism can lead to uncivil behavior in the workplace and psychological distress, which can increase employee procrastination (Malik, 2023). Moreover, Xiong and Zhang's (2023) study concluded that a leader’s perfectionism can hinder the development of innovative behaviors among employees. In contrast, this study proposes that a perfectionist leader can motivate subordinates to aim for high standards, thereby enhancing their work engagement and fostering radical innovations. This brings new insights into the study of a leader’s other-oriented perfectionism.

Third, most existing research concentrates on innovation outcome variables within organizations, such as employees’ innovative behaviors (Hoang et al., 2022; Lin et al., 2022). However, only a few studies have examined how an organization’s leadership traits affect the mechanisms of radical innovations. For organizations to thrive in a rapidly evolving external environment, their members must generate novel and groundbreaking ideas to distinguish themselves in intense competition. Consequently, the significance of employees’ radical innovative behaviors for organizational survival and growth is escalating daily and is invaluable in today’s volatile business landscape (Liu et al., 2022). This is why it is crucial to investigate the factors affecting radical innovation in organizations. This study considers a leader’s perfectionism as an influential factor and actively investigates the process of enhancing radical innovation.

Fourth, this study of Chinese small and medium-sized enterprises (SMEs) in the context of organizational culture with Chinese characteristics expands the research scope by incorporating both leadership traits and employee psychological states as moderating variables. Judge et al. (2002) argue that the Big Five personality traits are crucial for predicting leadership behavior. Therefore, this study considers conscientiousness as a leadership trait and examines its effect on employee behavior and states in roles that require perfectionism. Furthermore, this study incorporates the regulatory focus literature to explore differences in employees’ internal self-regulation toward radical innovations. Because individuals with a promotion focus tend to concentrate more on the positive outcomes of events, they are more likely to explore their environment and work (Wu and Zhang, 2023), which promotes radical innovation. Therefore, this study uses a promotion focus as a moderating variable to interact with work engagement to enhance radical innovation, thereby filling a research gap on this topic in Chinese SMEs.

## 2 Theoretical background

### 2.1 Leader’s other-oriented perfectionism and subordinate’s work engagement

This study uses Hewitt and Flett's (1991) concept of other-oriented perfectionism, which centers on expecting others to strive for perfection and being highly critical of those who fall short of expectations (Stoeber, 2015). According to Hewitt and Flett (1991), this kind of perfectionism is often maladaptive because it can lead to blaming, a lack of trust, and hostility, as well as setting unrealistic standards for others to set unrealistic standards, thus threatening the team climate (Kleszewski and Otto, 2020). However, some research suggests that this trait may have a ‘bright side’, whereby other-oriented perfectionism may help others to achieve high standards (Shoss et al., 2015). Leaders’ perfectionism toward their employees manifests itself in setting extremely high standards for their employees and requiring them to achieve their goals without flaws. If other-oriented perfectionism motivates individuals to help others meet standards, positive outcomes may result (Shoss et al., 2015). Drawing from trait interaction theory, research has demonstrated that other-oriented perfectionism in the workplace, when combined with a conscientious personality, can predict positive interpersonal outcomes at work, such as task-oriented helping behaviors (Shoss et al., 2015). They also propose that a leader’s perfectionism can positively affect employee creativity through job crafting (Yu et al., 2021).

Some other-oriented perfectionists may help others meet the high standards they hold, so when leaders set high standards while providing support and autonomy, employees are more likely to experience higher levels of engagement (Shoss et al., 2015). This engagement in turn motivates them to think creatively and pursue radical innovations. Thus, work engagement serves as a mediating mechanism that transforms the effects of leader perfectionism into a driver of innovation. Kahn (1990) defines engagement as the act of committing one’s physical, cognitive, and emotional resources to a work role. This engagement is evident when individuals are physically involved in a task, either individually or collectively, are cognitively alert and focused, and feel emotionally connected to their work. Engaged employees also enjoy exploring new ideas or actions, which can lead to more positive learning behaviors and proactive actions (Bakker et al., 2014). Work engagement is the perfect link that combines personal characteristics, work factors, and job performance, and is an important way to increase the competitive advantage of an organization (Guo and Hou, 2022). Job and personal resources have been found to predict work engagement, which in turn leads to improved job performance (Bakker, 2011). Additionally, findings based on a regression analysis indicate that work engagement acts as a mediator between perceived organizational support and employee creativity (Aldabbas et al., 2023).

A leader’s perfectionism can enhance the work engagement of their subordinates, because they are often motivated by the challenge of perfectionism and they view the leader’s emphasis on perfectionism as a significant learning opportunity, the high standards set by perfectionist leaders align with their desire to be challenged, leading to increased work engagement among employees working with such leaders (Xu et al., 2022). Moreover, a perfectionist leader can inspire subordinates to aim for high standards, thereby boosting their performance and efficiency (Ocampo et al., 2020). As a result, employees under a leader’s other-oriented perfectionism may enhance their engagement and performance at work to meet the expectations of perfectionist leaders (Birch et al., 2019). The high standards and demands of perfectionist leaders can stimulate positive emotions and the development of problem-focused coping strategies in employees, increasing their willingness to expend energy on work-related tasks and subsequently enhancing work engagement (Mazzetti et al., 2023). When employees perceive the perfectionist expectations of their leaders, they also feel the challenge of their work and simultaneously reap benefits and rewards. Overcoming this challenging pressure can yield positive effects and feedback for employees’ personal growth, leading them to value their work and invest more resources into it (Li et al., 2010). Therefore, a leader’s perfectionism, as a type of challenging stressor, sets high standards and requirements for employees, bringing pressure but also conveying positive ideas. This gives employees the confidence and passion to work hard, increasing their work commitment and subsequently improving their task performance.

Previous research by Amabile and Pratt (2016) suggests that creativity and innovation require the relentless pursuit of elevated standards, a focus on areas of concern, and the continuous motivation of employees to achieve their objectives. These elements can be effectively fostered by a perfectionist leader; therefore, a leader’s perfectionism could potentially enhance employees’ radical innovations by inspiring them to exert more effort and persist despite challenges and failures (Raza and Shah, 2023). A leader’s perfectionist demands inherently involve dissatisfaction with the current state of affairs and a quest for superior outcomes, to meet these demands, employees must deviate from existing solutions and explore different alternatives, thereby fostering creativity and enhancing radical innovation (Xu et al., 2022). Perfectionist leaders set high standards and expectations for their employees, when employees perceive these expectations, they infer that their leaders have strong faith in their abilities (Tierney and Farmer, 2004). This understanding motivates them to overcome their fear of challenging the status quo, ensuring optimal creative performance and increased radical innovation (Gong et al., 2009). Perfectionist leaders impose high standards and demands on their employees’ work. This compels employees to adjust their thinking, emotions, and other resources to reach the desired state when dealing with demanding tasks (Yu et al., 2021), and to seek new solutions that lead to radical innovations.

*H1:* Leader’s other-oriented perfectionism is positively related to subordinate’s work engagement.

*H2:* Leader’s other-oriented perfectionism is positively related to subordinate’s radical innovation.

### 2.2 Subordinate’s work engagement and subordinate’s radical innovation

Radical innovation is defined as one that has a strong impact on an organization, provides entirely new solutions and technologies that deliver benefits, and creates new business for the organization (O’Connor and Ayers, 2005). Radical innovation replaces old solutions and create entirely new ways of thinking that provide the engine for the long-term value growth of the business that business leaders seek and create new business opportunities (Leifer et al., 2001). This behavior embodies an employee’s ability to actively adopt innovative behaviors, courageously challenge the status quo, attempt new methods or creative thinking to solve technological issues, and potentially lead to disruptive innovations (Li et al., 2022). Therefore, radical innovation plays a crucial role in a firm’s economic sustainability (Koberg et al., 2003). Studies have shown that companies with a broad range of knowledge are more skilled at creating groundbreaking innovations when there is internal knowledge sharing (Zhou and Li, 2012). Leadership that supports innovation encourages employees to engage in radical innovation by fostering an identity tied to innovation (Liu et al., 2022). The values associated with pay raises, knowledge sharing, and thorough information processing all play multiple, sequential mediating roles in employees’ radical innovative behaviors (Yang and Wang, 2023).

When employees are deeply engaged in their work and less affected by external factors, they are better equipped to generate creative solutions and tackle problems innovatively, thereby boosting their creativity and fostering radical innovation (Wu et al., 2021). Employees who are highly engaged at work not only maximize the use of existing work resources, but also create new ones to sustain their engagement, as a result, these employees are more likely to work harder, be more efficient, exhibit greater creativity, and demonstrate a higher capacity for radical innovative behaviors (Bhatnagar, 2012). Furthermore, the sense of purpose that engaged employees feel at work encourages them to make extra efforts to understand problems from various angles and to connect with different information sources, which can promote radical innovation in the workplace (Gilson and Shalley, 2004; Montani et al., 2020). First, the positive emotional state linked with dedication in work engagement stimulates flexible thinking, which aids in generating creative solutions and enhancing radical innovation (Madrid et al., 2014). Employees who are engaged at work are fully immersed in their tasks, focusing on work-related activities and effectively using their resources (Chang et al., 2013). Additionally, work engagement allows employees to leverage their cognitive resources through absorption to discover new perspectives, information, and knowledge and integrate them into novel creative concepts (Zhang and Bartol, 2010). Work engagement empowers employees to fully use their resources, stimulate creative thinking, find new solutions and techniques, and enhance radical innovation.

*H3:* Subordinate’s work engagement is positively related to subordinate’s radical innovation.

### 2.3 The mediating effect of subordinate’s work engagement

Perfectionist leaders drive employees to pursue higher performance by setting high expectations and striving for excellence, emphasizing the gap between the current and ideal state (Mitchell et al., 2019; Park et al., 2014). This behavior motivates employees to invest more physical, cognitive, and emotional resources to enhance work engagement (Xu et al., 2022). Work engagement enables employees to actively engage in cognitive activities, absorb new knowledge, and drive radical innovation (Parker and Griffin, 2011). Thus, leadership perfectionism promotes employees’ self-regulation by motivating them to align with organizational goals, ultimately enhancing innovation. Research has shown that perfectionist leaders enhance employees’ sense of efficacy and work engagement by setting high standards and clear expectations (Tierney and Farmer, 2011). Engaged employees are more inclined to seek out learning opportunities, develop expertise, and generate innovative ideas (Park et al., 2014). In addition, employees respond to high demands by increasing their work engagement, both to satisfy intrinsic needs and to achieve innovative outcomes (Yu et al., 2021). Organizations can leverage leadership perfectionism to promote innovation through a supportive environment. For example, leaders align employees with organizational goals through effective communication, boosting their confidence and motivation (Hakanen et al., 2008). By enhancing work engagement, organizations can transform the pressure of leadership perfectionism into a drive for radical innovation, achieving a win-win situation for both employees and the organization.

*H4:* Subordinate’s work engagement positively mediates the relationship between leader’s other-oriented perfectionism and subordinate’s radical innovation.

### 2.4 The moderation effects of leader’s conscientiousness

Conscientiousness refers to a collection of constructs that describe individual differences in tendencies to exhibit self-control, responsibility toward others, diligence, organization, and adherence to rules (Roberts et al., 2009). Individuals with high conscientiousness typically organize their time, work in a disciplined way toward their objectives, aim for precision and perfection in their tasks, and deliberate carefully when making decisions (Smithikrai and Suwannadet, 2018). Conscientious leaders can significantly aid employees in overcoming anxiety and insecurity, enhancing subjective wellbeing, and responding to the pressing need to tackle the challenges of the new work-life environment (Xue et al., 2023). Conscientious leaders boost performance by aiding in the establishment of norms and behaviors that ultimately inspire employees to actively engage in work process improvement (Walumbwa et al., 2012). Xue et al. (2023) indicate that leaders who exhibit higher levels of conscientiousness may enhance the wellbeing of their subordinates. Smithikrai and Suwannadet (2018) suggest that conscientiousness is a moderating factor in the direct relationship between authentic leadership and proactive work behavior, with the relationship being stronger when the leader’s conscientiousness is high. Wang et al. (2020) found that a leader’s conscientiousness moderates the indirect effect of a leader’s humility on promoting team creativity through team creativity effectiveness.

Leaders who are perfectionists establish high standards and expectations for others. In this study, we explore the personality traits associated with other-oriented perfectionism and examine how a leader’s conscientiousness can enhance employee work engagement. A perfectionist leader often favors the performance of others, but the level of their conscientiousness leads to the preference can be successfully converted into supportive behaviors that boost employee work engagement (Shoss et al., 2015). Perfectionistic leaders have high expectations of their employees, when these leaders are also conscientious, they engage in socially acceptable behaviors, adhere to ethical standards and rules, and hold themselves accountable to their employees, this accountability helps keep employees engaged and flexible in completing their tasks (Van Eeden et al., 2008). Conscientious individuals, who are often perfectionists, perform their work meticulously to avoid errors and make informed decisions, these leaders motivate their followers by setting high yet achievable goals and providing assistance when needed; therefore, when employees have a leader who is both a perfectionist and conscientious, they receive the necessary leadership support to fully engage in their work (Breevaart and de Vries, 2021). According to the job demands-resources (JD-R) model, job demands and job resources can act as precursors to employee work engagement, reducing job demands helps employees concentrate on their work and minimize unproductive time, while increasing job resources helps employees maintain their energy and stay engaged in their work, if sufficient job resources are available, they can counteract the negative effects of demands, thereby ensuring high levels of work engagement and subsequent positive outcomes (Bakker and Demerouti, 2007). Therefore, a leader’s perfectionism, which places high demands on employees and challenges them to promote desirable behaviors, raises the standard of work and provides employees with the necessary work resources to stay engaged in their work and perform relevant tasks effectively under the personality trait of a leader’s conscientiousness.

*H5:* Leader’s conscientiousness positively moderates the relationship between leader’s other-oriented perfectionism and subordinate’s work engagement.

### 2.5 The moderated mediation effects of subordinate’s promotion focus

The regulatory focus principle differentiates between self-regulation with a promotion focus, which concerns achievement and ambition, and self-regulation with a prevention focus, which concerns safety and responsibility. When individuals have a promotion focus, they are motivated to seek pleasure, and this focus is linked to the motivation to reach a desired outcome (Higgins, 1997). People engaged in a promotion-focused self-regulatory process are motivated by their growth and developmental needs to strive to align with their ideal selves, thereby enhancing the importance of positive outcomes (Brockner and Higgins, 2001). Individuals with a promotion focus tend to view the environment as benign and use a variety of strategies to achieve their goals (Zhou et al., 2012). When people have a promotion focus, they prefer to gain new achievements rather than maintain current ones, value goals that involve accomplishments or outcomes perceived as gains, and persist in tasks that promise rewards for success (Molden et al., 2009). Transformational leadership has a positive effect on employee creativity, facilitated by the mediating effects of promotion focus (Henker et al., 2015). Furthermore, transformational leadership proves particularly beneficial for job crafting when employees have a high promotion focus (Hetland et al., 2018). Challenging stress positively affects promotion focus, which in turn positively affects creativity self-efficacy (Wu et al., 2021).

We emphasize the significant moderating role of an employee’s promotion focus in amplifying the effect of work engagement on radical innovation. Essentially, radical innovation is shaped by the interplay between work engagement and promotion focus. Employees with a promotion focus are likely to be more open to risk-taking and experimentation with creative strategies, and more driven to implement creative ideas, leading to radical innovations (Henker et al., 2015). Such employees foster positive changes in their work, enhancing their work resources and fostering work engagement, boosting positive emotions, new work perceptions, and the emergence of radical innovations (Lichtenthaler and Fischbach, 2019). Employees with a promotion focus are motivated to engage in their work to fulfill their aspirations and concentrate on positive outcomes (Blank and Naveh, 2018). This process supports creative insights (Friedman and Förster, 2001), enabling employees to explore new knowledge and develop new competencies, leading to radical innovation. Regulatory focus theory suggests that an individual’s motivation aids in achieving their desired end state, driven by this growth desire, employees with a promotion focus engage in work to realize their accomplishments, explore new work solutions, and achieve radical innovations (Jason and Geetha., 2021). Based on these arguments, employees with a promotion focus believe in their ability to perform their jobs well and dedicate their full mental and physical energy to their work. This leads to the acquisition of new knowledge and skills, the generation of new work options, and the achievement of certain accomplishments, resulting in more radical innovation. This process suggests that high levels of employee promotion focus and work engagement foster high levels of radical innovation.

Moreover, employees who are intrinsically motivated and have a promotion focus tend to be more engaged in their work and seek innovative solutions to problems, leading to innovation (Yidong and Xinxin, 2013). High expectations from a perfectionist leader enhance these employees’ cognitive flexibility, risk-taking, and confidence in complex tasks, thereby generating new ideas and creative solutions, and enhancing radical innovation (Grant and Berry, 2011). Therefore, this study concludes that an employee’s work engagement is linked to their psychological state of promotion focus when their leader exhibits perfectionist traits, and this can affect radical innovation. Employees with a promotion focus are more attuned to the high work standards set by their leaders and, as a result, they seek innovative ways to structure their work and embrace the challenge of working toward the leader’s vision (Hetland et al., 2018). Thus, when faced with their leader’s perfectionist expectations, employees with a promotion focus can increase their work engagement, seek new ways of working, and achieve radical innovation. When perfectionist leaders set high standards for work engagement, employees adjust their behavior to actively engage in their work, when this high level of work engagement is paired with the stimulation of high work demands, employees with a promotion focus find the most favorable work environment, strive to perform creative work, and achieve radical innovation (Zhou et al., 2012). In general, when employees have higher levels of promotion focus, the high work demands associated with leader perfectionism are more likely to be viewed as a challenging stressor, perceived as contributing to personal growth and development, and enhancing personal work engagement, this, in turn, motivates employees to develop positive emotions and coping behaviors in response to stress and employees with a promotion focus are more open-minded, innovative, and willing to take risks, contributing to radical innovation (Yu et al., 2021). Therefore, this study concludes that when employees have a higher promotion focus, they are more willing to work hard, explore new solutions, and the higher their work engagement, the greater their radical innovation. Furthermore, when faced with the high demands and standards of a leader’s perfectionism, employees with a promotion focus have a more open attitude toward challenges, a higher willingness to take risks, and positive emotions toward challenges, and are more likely to be inspired to make radical innovations.

*H6:* Subordinate’s promotion focus will positively moderate the relationship between subordinate’s work engagement and subordinate’s radical innovation. Such that when the level of subordinate’s promotion focus is higher, the positive relationship of subordinate’s work engagement on subordinate’s radical innovation is enhanced.

*H7:* The mediating role of subordinate’s work engagement on the relationship between leader’s other-oriented perfectionism and subordinate’s radical innovation will be positively moderated by subordinate’s promotion focus.

## 3 Method

### 3.1 Participants and procedures

To empirically analyze the research hypothesis that leader other-oriented perfectionism is an antecedent of radical innovation, we surveyed employees of Chinese SMEs through an online questionnaire. The majority of employees who participated in the survey were subordinates. The sample size of the recovered questionnaires for analysis was 343 (91.2% response rate), after excluding invalid questionnaires. Regarding the characteristics of the participants in this study, there were 134 men (39.1%) and 209 women (60.9%). In terms of age, 0 (0%) were under the age of 20 years, 62 (18.1%) were between the ages of 20 and 29 years, 71 (20.6%) were between the ages of 30 and 39 years, 110 (32.1%) were between the ages of 40 and 49 years, 100 (29.2%) were between the ages of 50 years or older. In terms of educational level, 111 (32.4%) were from technical secondary school or high school, 72 (21.0%) were from junior college, 84 (24.4%) graduated from college, 17 (5.0%) held a master’s degree, 3 (0.9%) held a doctor’s degree or higher and 56 (16.3%) are in other. In terms of employment relationships, full-time jobs were the most numerous at 213(62.1%) and informal positions were 130(37.9%).

Regarding Service Years, 29(8.5%) people had worked for a year or under, 42(12.2%) had worked for 1 to 3 years, 42(12.2%) had worked for 3 to 5 years, 37(10.8%) had worked for 5 to 7 years, and 193(56.3%) people had worked for 7 or over. Regarding about the time to work with the current immediate leader, 51(14.9%) people had worked for a year or under, 41(12.0%) had worked for 1 to 2 years under, 50(14.6%) had worked with the current immediate leader for 2 to 3 years under, 31(9.0%) had worked with the current immediate leader for 3 to 4 years under, 32(9.3%) worked with the current immediate leader for 4 to 5 years under, and 138(40.2%) people had worked with the current immediate leader for 5 or over. Regarding enterprise type, 27(7.9%) people were working in education, 37(10.8%) people were working in finance, 25(7.3%) people were working in medical industry, 71(20.7%) people were working in catering services, 36(10.5%) people were working in coal mining, 138(40.2%)people were work in media and 9(2.6%) people were working in other occupations.

### 3.2 Measures

Leader other-oriented perfectionism. To measure leader other-oriented perfectionism in Chinese SMEs, we used Hewitt and Flett (1991) measurement scale. The measurement tool consists of 5 items. The sample items included,“My leaders have great expectations for me.” and “My leader expects me to do my job perfectly.”

Work engagement. To measure the work engagement of Chinese SME members, we used a measurement scale consisting of 18 questions from Rich et al. (2010). The sample items include “I work with intensity on my job.” and “I exert my full effort to my job.”

Conscientiousness. To measure leader conscientiousness in Chinese SMEs, we used Gerlitz and Schupp (2005) measurement scale. The measurement tool consists of 5 items. The sample items included, “My leader is an organized person.” and “My leader is a responsible person.”

Promotion focus. This study used the measurement items of Wallace and Chen (2006) to measure promotion focus. The measurement tool consists of 6 items. Sample items include “I can accomplish a lot at work.” and “I’ll do my job well no matter what.”

Radical innovation. We used Li et al. (2008) scale to measure Chinese SMEs’ radical innovation. The tool used to measure radical innovation consists of 4 items. Sample items include “I often create radically new products.” and “I often introduce radically new concept in innovations.”

**Figure 1:**
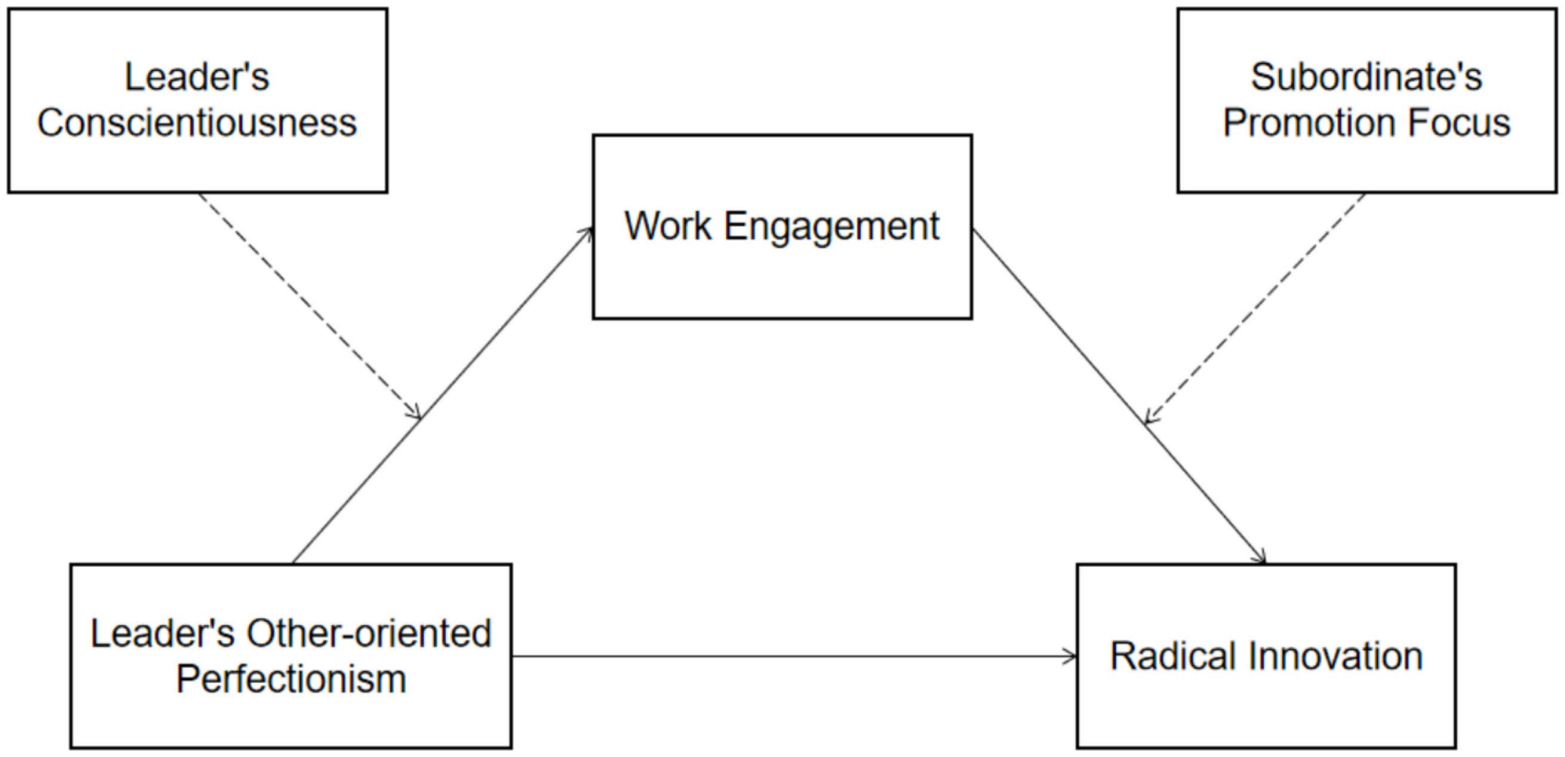
The research model.

All items use a 7-point Likert scale, with responses ranging from 1 (strongly disagree) to 7 (strongly agree); the higher the score, the stronger the abovementioned intent. The research model is shown in Figure 1.

### 3.3 Analytical approach

The collected questionnaire data were statistically analyzed using SPSS 26.0. and Amos 24.0. Initial data analysis was completed using SPSS software, covering demographic characterization, reliability analysis of the scales (assessed by Cronbach’s alpha coefficients), and correlation analyses between variables. Subsequently, confirmatory factor analysis (CFA) was conducted using AMOS software to test the structural validity of the measurement model. In this study, SPSS Process Macro 3.4.1 Model 4 was used to analyze the direct, indirect, and mediating effects of the variables. In order to test the robustness of the moderating effect, this study used the SPSS PROCESS Macro 3.4.1 Model 1 to conduct 5,000 samples of Bootstrapping tests within the 95% confidence interval. Finally, this study validated the overall model using structural equation modeling in SmartPLS software.

## 4 Results

### 4.1 Confirmatory factor analysis and reliability analysis

This study first conducted a confirmatory factor analysis. Confirmatory factor analysis (CFA) is a type of structural equation modeling that deals specifically with measurement models; that is, the relationships between observed measures or indicators (e.g., test items, test scores, behavioral observation ratings) and latent variables or factors (Brown and Moore, 2012). Model 1 was an expected model, in which five factors were loaded independently and input simultaneously. In terms of model fit, the absolute fit index was *X*^2^(*p*) = 661.538(0.000), *X*^2^/df = 1.010, RMSEA = 0.050, and the incremental fit index was IFI = 0.941, CFI = 0.940, and the parsimonious adjusted index was PGFI = 0.806, PNFI = 0.876. Model 2 was designed using all items loaded on a single factor. The results showed X^2^(*p*) = 4625.311(0.000), X^2^/df = 6.955, RMSEA = 0.132, IFI = 0.621, CFI = 0.619, PNFI = 0.552, and PGFI = 0.434. Based on these results, we acknowledge that Model 1 is acceptable with a good fit. Table 1 summarizes the results of the structural model fit index.

**Table 1:** Summary of structural model fit results.

Model	χ^2^ (*p*)	χ^2^ /df	RMSEA	IFI	CFI	PNFI	PGFI
Model 1 (Expected Model of five-factor^a^)	661.538	1.010	0.050	0.941	0.940	0.876	0.806
Model 2 (one-factor^b^)	4625.311	6.955	0.132	0.621	0.619	0.552	0.434

^aLeader Other-oriented perfectionism, work engagement, conscientiousness, promotion focus, and radical innovation; bAll items were loaded on a single factor.^

The CFA of Model 1 (five-factor model) showed that the scale was a good fit and construct validity. To verify the feasibility of the model, we derive the Average Variance Extracted (AVE) and Composite Reliability (CR). In terms of the AVE value, all the values are greater than 0.5. Regarding the value of CR, all the values are greater than 0.7. Through such a result, convergent validity is ensured. Refer to Table 2 for specific values.

**Table 2:** The result of convergent validity and reliability analysis.

Variables		Estimate	S.E.	C.R.	*p*	Standardized regression weights	AVE	C.R	Cronbach’s alpha
Leader other-oriented perfectionism (A)	A1	1				0.726	0.649	0.894	0.902
	A2	0.862	0.058	14.903	***	0.832			
	A3	0.811	0.055	14.814	***	0.827			
	A4	0.835	0.057	14.601	***	0.815			
	A5	0.801	0.054	14.729	***	0.823			
Work engagement (B)	B1	1				0.843	0.684	0.975	0.975
	B2	0.705	0.036	19.554	***	0.827			
	B3	0.699	0.036	19.380	***	0.822			
	B4	0.682	0.036	18.855	***	0.809			
	B5	0.675	0.035	19.417	***	0.823			
	B6	0.707	0.036	19.591	***	0.828			
	B7	0.699	0.036	19.194	***	0.817			
	B8	0.741	0.037	20.076	***	0.84			
	B9	0.727	0.036	20.157	***	0.842			
	B10	0.692	0.035	19.512	***	0.826			
	B11	0.712	0.037	19.454	***	0.824			
	B12	0.710	0.036	19.481	***	0.825			
	B13	0.702	0.037	19.148	***	0.816			
	B14	0.731	0.037	19.670	***	0.83			
	B15	0.681	0.035	19.195	***	0.818			
	B16	0.716	0.036	20.081	***	0.84			
	B17	0.741	0.037	19.986	***	0.837			
	B18	0.729	0.038	19.371	***	0.822			
Conscientiousness (C)	C1	1				0.81	0.681	0.912	0.914
	C2	0.746	0.042	17.671	***	0.836			
	C3	0.738	0.042	17.622	***	0.835			
	C4	0.748	0.043	17.231	***	0.821			
	C5	0.749	0.043	17.346	***	0.825			
Promotion focus (D)	D1	1				0.79	0.693	0.928	0.931
	D2	0.827	0.046	18.030	***	0.861			
	D3	0.800	0.045	17.909	***	0.857			
	D4	0.781	0.045	17.909	***	0.832			
	D5	0.772	0.045	17.233	***	0.832			
	D6	0.767	0.045	16.924	***	0.821			
Radical innovation (E)	E1	1				0.772	0.667	0.883	0.889
	E2	0.813	0.052	15.767	***	0.828			
	E3	0.826	0.053	15.715	***	0.825			
	E4	0.821	0.051	16.017	***	0.84			
Model fit index		X^2^(*p*) = 661.538(0.000), X^2^/df = 1.010, RMSEA = 0.050, IFI = 0.941, CFI = 0.940, GFI = 0.912, RMR = 0.072, PGFI = 0.806, PNFI = 0.876, SRMR = 0.030							

The reliability analysis results of each variable in this study are all above 0.7, thus ensuring confidence in each variable. Table 2 shows the results of the analysis of convergent validity and reliability.

### 4.2 Descriptive statistics and correlation analysis

In this study descriptive statistical analyses included mean and standard deviations. The means, standard deviations and correlations of the variables are in accordance with the criteria. Table 3 shows the results of the descriptive statistics and correlation analysis.

**Table 3:** The results of descriptive statistics and correlation analysis.

Variables	Mean	SD	Leader other-oriented perfectionism	Work engagement	Conscientiousness	Promotion focus	Radical innovation
Leader other-oriented perfectionism	4.599	1.256	1				
Work engagement	4.429	1.223	0.436^***^	1			
Conscientiousness	4.546	1.263	0.262^***^	0.376^***^	1		
Promotion focus	4.301	1.345	0.142^***^	0.147^***^	0.082	1	
Radical innovation	4.098	1.366	0.250^***^	0.282^***^	0.189^***^	0.291^***^	1

^***^p < 0.001; ^**^p < 0.01; ^*^p < 0.05.

### 4.3 Hypothesis test

The results show that work engagement of employees in Chinese SMEs has a mediating effect on the relationship between leader other-oriented perfectionism and radical innovation. The results of the analysis are as follows: leader other-oriented perfectionism had positive effects on work engagement (*t* = 8.624, *p* < 0.001), and Boot LLCI was found to be 0.314 and Boot ULCI was found to be 0.496, which did not contain 0, so hypothesis 1 was supported. Work engagement had positive effects on radical innovation (*t* = 3.887, *p* < 0.001), and Boot LLCI was found to be 0.108 and Boot ULCI was found to be 0.325, which did not contain 0, so hypothesis 3 was supported. Leader other-oriented perfectionism had positive effects on radical innovation (*t* = 0.2.409, *p* < 0.05), and Boot LLCI was found to be 0.027 and Boot ULCI was found to be 0.235, which did not contain 0, so hypothesis 2 was supported.

In this study, SPSS Process Macro 3.4.1 Model 4 was used to test for mediating effects, and 5,000 Bootstrap samples were taken for 95% confidence interval estimation, and the results of the test are shown in the Table 4. The indirect effect of work engagement in the relationship between leader other-oriented perfectionism and radical innovation is 0.087, with a lower limit of 0.043 and an upper limit of 0.139. The display does not contain 0 between the upper and lower values, and therefore the effect of the parameter can be considered significant. Hence, hypothesis 4 is supported. Table 4 shows the results.

**Table 4:** The results of mediating effect.

Path			Estimate	S.E.	*t*	*p*	LLCI	ULCI
Leader Other-oriented perfectionism	→	Work engagement	0.402	0.047	8.624	0.000	0.314	0.496
Work engagement	→	Radical innovation	0.217	0.056	3.887	0.000	0.108	0.325
Leader other-oriented perfectionism	→	Radical innovation	0.130	0.054	2.409	0.016	0.027	0.235
Total effect of X on Y								
Leader other-oriented perfectionism → Work engagement → radical innovation			0.217	0.049	4.457	0.000	0.123	0.314
Direct effect(s) of X on Y								
Leader other-oriented perfectionism → radical innovation			0.130	0.054	2.409	0.016	0.027	0.235
Indirect effect(s) of X on Y								
Leader other-oriented perfectionism → work engagement → radical innovation			0.087	0.024	3.576	0.000	0.043	0.139

**Figure 2:**
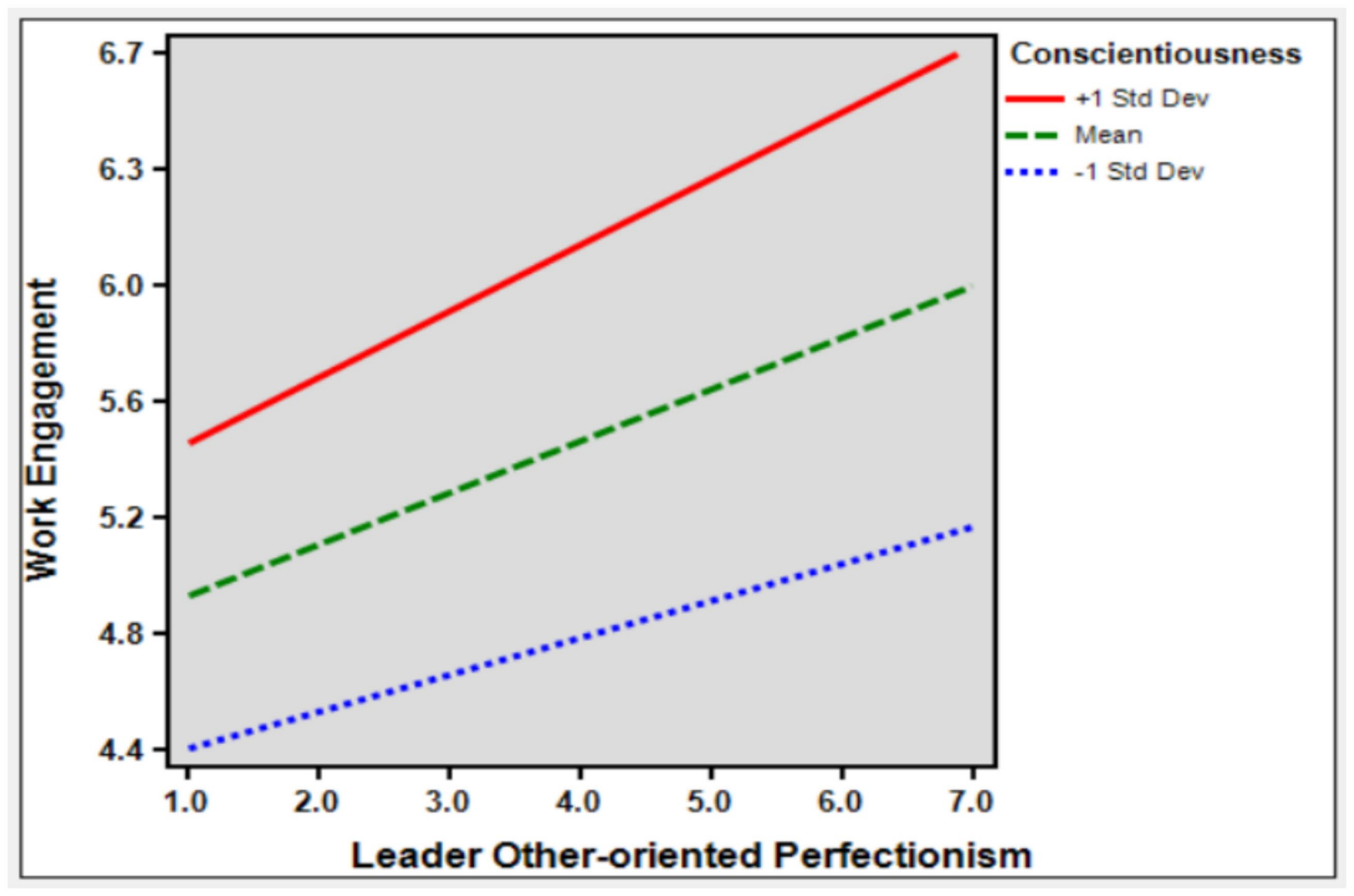
The moderating effect of conscientiousness.

Leader other-oriented Perfectionism was set as the independent variable, Work Engagement was set as the dependent variable and Leader’s Conscientiousness was set as the moderating variable for moderating effect analysis. From the effect coefficients in the above table, it can be seen that the regression coefficient between Leader other-oriented Perfectionism and Work Engagement is 0.402 and the significance level is *p* < 0.001 level, so there is a significant positive effect between Leader other-oriented Perfectionism and Work Engagement has a significant positive effect relationship and the regression coefficient of the interaction term (Leader other-oriented Perfectionism*Leader’s Conscientiousness) is 0.187 and the level of significance *p* < 0.001 level, so Leader’s Conscientiousness has a positive moderating role in the effect of Leader other-oriented Perfectionism on Work Engagement. Therefore, hypothesis 5 is supported. Figure 2 shows the graph related to the moderating effect of conscientiousness (Table 5).

**Table 5:** The result of moderation.

Variable	Estimate	S.E.	*t*	*p*	95% CI	
Leader other-oriented perfectionism (A)	0.402	0.047	8.624	0.000	0.314	0.496
Leader’s conscientiousness (B)	0.322	0.047	6.839	0.000	0.232	0.417
Interaction (A*B)	0.187	0.044	4.212	0.000	0.101	0.275

^**^p < 0.001; ^**^p < 0.01; ^*^p < 0.05.

**Figure 3:**
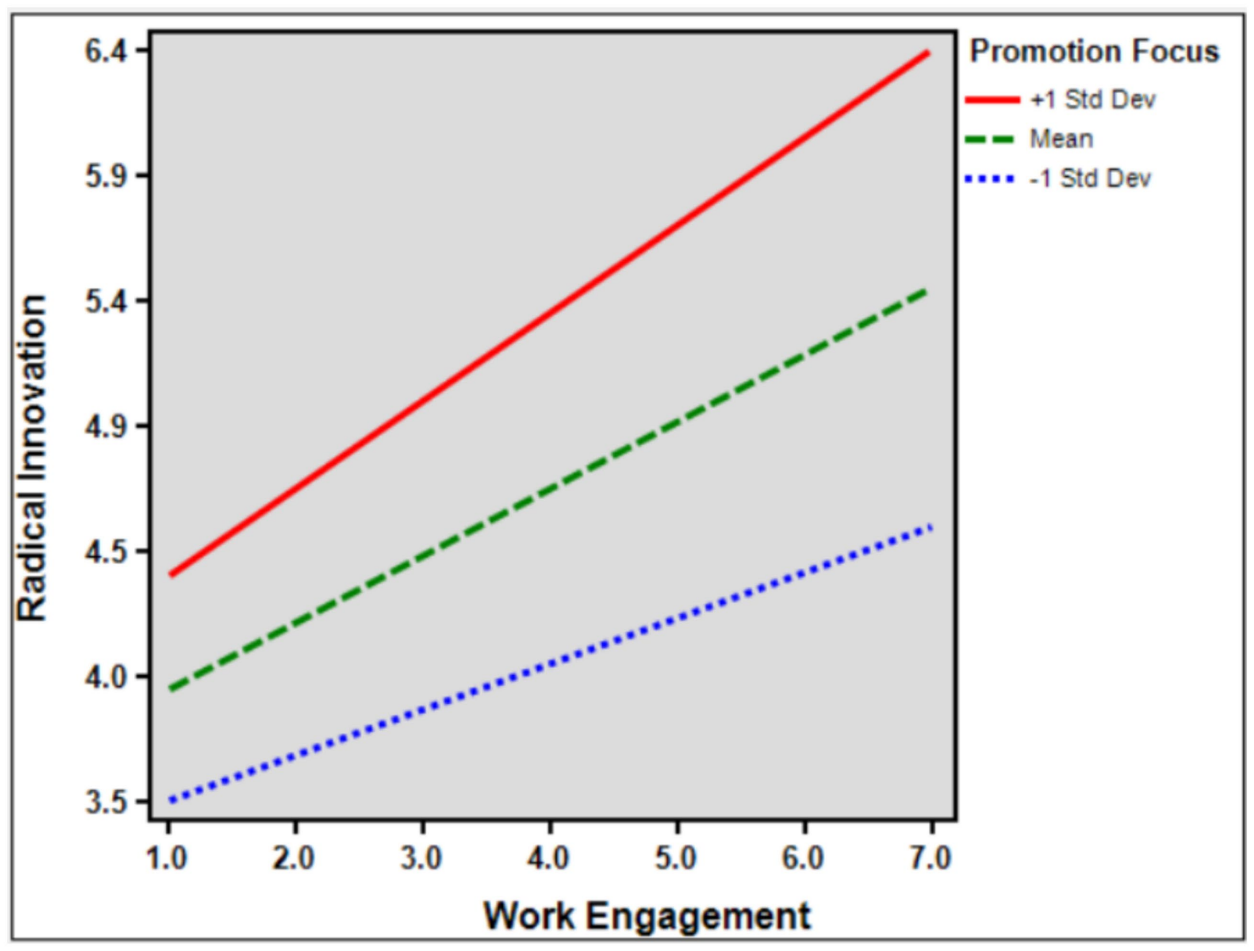
The moderating effect of promotion focus.

Work Engagement was set as the independent variable, Radical Innovation as the dependent variable and Subordinate’s Promotion Focus as the moderating variable for moderating effect analysis. From the effect coefficients in the above table, it can be seen that the regression coefficient between Work Engagement and Radical Innovation is 0.217, and the significance level is *p* < 0.001 level, so there is a significant positive effect relationship between Work Engagement and Radical Innovation, and the interaction terms (Work Engagement*Subordinate’s Promotion Focus) has a regression coefficient of 0.185 and a significance level of *p* < 0.001 level, so there is a significant positive effect relationship between Work Engagement and Radical Innovation, and the interaction term (Work Engagement*Subordinate’s Promotion Focus) has a positive moderating effect in the effect of Work Engagement on Radical Innovation. There is a positive moderating effect in the effect of Work Engagement on Radical Innovation. Therefore, hypothesis 6 is supported. Figure 3 shows the graph related to the moderating effect of promotion focus (Table 6).

**Table 6:** The result of moderation.

Variable	Estimate	S.E.	*t*	*p*	95% CI	
Work engagement (A)	0.217	0.056	3.887	0.000	0.108	0.325
Subordinate’s promotion focus (B)	0.242	0.050	4.826	0.000	0.146	0.344
Interaction (A*B)	0.185	0.053	3.514	0.000	0.083	0.289

^**^p < 0.001; ^**^p < 0.01; ^*^p < 0.05.

This study used SmartPLS software to conduct hypothesis testing for structural equation modeling. The fit of the initial research model was first examined. In terms of model fit, the absolute fit index was *X*^2^(*p*) = 663.473(0.000), *X*^2^/df = 1.013, RMSEA = 0.060, SRMR = 0.035, and the incremental fit index was IFI = 0.940, CFI = 0.940, and the results of the examination found that the model fit was good, indicating that the fit of the structural model was acceptable.

This study follows the division criteria of mean plus or minus one standard deviation to test whether there is a difference in the mediating effect of Work Engagement on the impact of Leader other-oriented Perfectionism on Radical Innovation at two different levels of Subordinate’s Promotion Focus, low and high.

The results of the analysis of hypothesis 7 are shown in Table 7. Based on the test results, when Leader’s Conscientiousness is held constant as the reference group, we examined the mediating effect of Work Engagement between Leader Other-Oriented Perfectionism and Radical Innovation at different levels of Subordinate’s Promotion Focus. The results indicate that the mediating effect size of Work Engagement varies depending on the level of Subordinate’s Promotion Focus. When Leader’s Conscientiousness is at a lower level (M-1SD) and Subordinate’s Promotion Focus is at a lower level (M-1SD), the mediated effect of Work Engagement has a value of 0.008, with a significance *p*-value of 0.626 which is greater than the significance threshold. Level 0.05 and the 95% confidence interval contains 0, so its indirect effect is not significant; when Subordinate’s Promotion Focus is at a higher level (M + 1SD), the mediation effect of Work Engagement is 0.085, with a significance p-value of 0.008 which is smaller than the significance threshold level of 0.05 and 95% confidence interval does not contain 0, so its indirect effect is significant. When Leader’s Conscientiousness is at a Mean level and Subordinate’s Promotion Focus is at a lower level (M-1SD), the mediated effect of Work Engagement has a value of 0.015, with a significance p-value of 0.610 which is greater than the significance threshold level 0.05 and the 95% confidence interval contains 0, so its indirect effect is not significant; when Subordinate’s Promotion Focus is at a higher level (M + 1SD), the mediation effect of Work Engagement is 0.166, with a significance p-value of 0.000 which is smaller than the significance threshold level of 0.05 and 95% confidence interval does not contain 0, so its indirect effect is significant. When Leader’s Conscientiousness is at a higher level (M + 1SD) and Subordinate’s Promotion Focus is at a lower level (M-1SD), the mediated effect of Work Engagement has a value of 0.022, with a significance p-value of 0.610 which is greater than the significance threshold level 0.05 and the 95% confidence interval contains 0, so its indirect effect is not significant; when Subordinate’s Promotion Focus is at a higher level (M + 1SD), the mediation effect of Work Engagement is 0.246, with a significance p-value of 0.000 which is smaller than the significance threshold level of 0.05 and 95% confidence interval does not contain 0, so its indirect effect is significant. This suggests that there is a moderated mediating effect in this study, i.e., Subordinate’s Promotion Focus moderates the mediating effect of Work Engagement between Leader other-oriented Perfectionism and Radical Innovation, and as Subordinate’s Promotion Focus increases, the mediating effect of Work Engagement between Leader other-oriented Perfectionism and Radical Innovation increases, i.e., Subordinate’s Promotion Focus positively moderates the mediating effect of Work Engagement between Leader other-oriented Perfectionism and Radical Innovation. So, hypothesis H7 is supported (Table 7).

**Table 7:** The moderated mediation effect of promotion focus.

Dependent variable: radical innovation								
Leader’s conscientiousness	Moderator	Level	Indirect effect	SE	*t*	*p*	95% CI	
−1 SD	Promotion focus	−1 SD	0.008	0.016	0.487	0.626	−0.022	0.041
		M	0.046	0.019	2.483	0.013	0.015	0.088
		+1 SD	0.085	0.032	2.643	0.008	0.030	0.155
Mean	Promotion focus	−1 SD	0.015	0.029	0.510	0.610	−0.042	0.073
		M	0.090	0.027	3.373	0.001	0.041	0.147
		+1 SD	0.166	0.043	3.818	0.000	0.089	0.260
+1 SD	Promotion focus	−1 SD	0.022	0.043	0.510	0.610	−0.062	0.108
		M	0.134	0.040	3.318	0.001	0.061	0.220
		+1 SD	0.246	0.066	3.753	0.000	0.129	0.389

## 5 Discussion

This study concentrates on employees of Chinese SMEs, specifically investigating how a leader’s perfectionism predicts employee radical innovation. It tests the mediating effect of employee work engagement and the moderating effects of a leader’s conscientiousness and employee promotion focus on the relationship between a leader’s perfectionism and radical innovation. Based on the results, this study found that work engagement mediating the link between leader perfectionism and radical innovation, with leader conscientiousness and promotion focus moderating the effects.

### 5.1 Theoretical implications

First, this study’s findings indicate that leadership perfectionism positively is an antecedent of employee work engagement. This implies that an increase in a leader’s perfectionism corresponds to an increase in the level of employee work engagement. This contradicts Xiong and Zhang's (2023) conclusion that a leader’s perfectionism leads to employee burnout. However, it is in line with the findings of Ocampo et al. (2020). Leaders’ perfectionism inherently motivates employees because of their ability to facilitate the mastery of knowledge by meeting basic needs (Mazzetti et al., 2023). Therefore, a leader’s perfectionism predicts employees’ resilience by coaching and motivating them, promoting task-related knowledge and strategies, and affecting their work engagement to achieve organizational objectives.

Second, there is a positive correlation between employee work engagement and radical innovation. This aligns with the findings of Gupta et al. (2017), which suggest that employees with high work engagement are more likely to exhibit innovative behavior. Adopting innovative work practices requires a significant amount of work engagement. Radical innovation, which involves devising new solutions, requires employees to be focused, mentally resilient to resist distractions, and feel satisfied with their work, dedicating their time and energy to it (Agarwal, 2014). Therefore, this study concludes that employees who are fully engaged in their work are more likely to enhance their knowledge and skills and stimulate flexible thinking, which in turn leads to radical innovation.

Third, the mediating effect of employee work engagement is evident in the positive path of a leader’s perfectionism influencing employee radical innovation. A leader’s perfectionism may establish more stringent performance standards or elevate organizational goals for employees (Ocampo et al., 2020). According to self-regulation theory, a leader’s perfectionism triggers the self-regulatory behavior of job crafting, which subsequently boosts employees’ self-efficacy and intrinsic motivation for creativity, thereby fostering creativity (Yu et al., 2021). Therefore, a leader’s perfectionism can serve as a motivational tool to ignite employees’ intrinsic motivation, enhance work engagement, and ultimately improve their radical innovation significantly.

Fourth, the positive role of a leader’s perfectionism on employee work engagement is moderated by the leader’s conscientiousness. This study found that the higher the leader’s conscientiousness, the stronger the effect of the leader’s perfectionism on employee work engagement. This is in line with the findings of Shoss et al. (2015). Conscientious leaders maintain order, behave in a socially acceptable way, and are responsible for their employees and their work until the task is completed (Van Eeden et al., 2008). The high-performance traits of conscientious leaders also lead them to provide more resources, effectively enabling employees to increase their work engagement and successfully complete work-related tasks (Shoss et al., 2015). Therefore, while a leader’s perfectionism sets high standards and demands, conscientiousness drives leaders to support employees with the resources they need to perform their jobs. When employees receive this support, their work engagement increases significantly.

Lastly, with high levels of leader conscientiousness and high levels of employee promotion focus, the mediating effect of work engagement in the pathway of perfectionism positively influencing radical innovation will be enhanced. Leaders with high levels of conscientiousness not only take responsibility for work outcomes, but also proactively provide employees with the resources and support they need to accomplish their tasks (Shoss et al., 2015). This support, which includes clear guidance, necessary tools, and emotional encouragement, can help employees better cope with the high standards and demands set by perfectionist leaders. Promotion focus individuals are more inclined to adopt exploratory behaviors and try new approaches and strategies when faced with complex or uncertain tasks (Zhou et al., 2012). This exploratory behavior not only enhances their work engagement, but also provides them with more opportunities for innovation. Therefore, when leaders have a high level of conscientiousness and at the same time employees have a high level of promotion focus, the two have a synergistic effect. Conscientiousness leaders provide the necessary support and resources to their employees, while promotion focus employees take full advantage of these resources to actively explore and innovate. This synergy significantly enhances the mediating effect of work engagement in the pathway to perfectionism facilitates radical innovation.

The primary contribution of this study is the introduction of a research model that draws on previous studies on a leader’s perfectionism, conscientiousness, employee work engagement, promotion focus, and radical innovation, suggesting correlations and implications among these variables. It also challenges the prevalent belief that the personality trait of other-oriented perfectionism results in negative outcomes. In contrast to prior research findings, this study asserts that a leader’s perfectionism can also yield positive results, such as employee engagement and radical innovation. Therefore, it offers a fresh viewpoint that is insightful in understanding how a leader’s perfectionism promotes employee radical innovation.

### 5.2 Practical implications

The research of Xu et al. (2022) findings suggest that perfectionism has a double-edged effect on employee creativity. Perfectionism has a double-edged effect to foster creativity, leaders must manage their level of perfectionism carefully to mitigate its negative effects. When perfectionism becomes extreme, its beneficial effect can diminish or disappear. Before setting goals, leaders should communicate with employees about their capabilities and resources to ensure that the goals are both challenging and within their capabilities. Provide employees with dedicated resource support for attempting innovative projects that are high-risk but potentially high-reward. Set up an innovation incentive program to reward employees who come up with innovative ideas or successfully implement innovative projects with material or spiritual rewards. In conclusion, to prevent the harmful desire for control due to an excessive pursuit of perfection, leaders should reduce their excessive supervision of employees, provide sufficient space for creativity, strengthen the employees’ innovation role identity within the organization, and nurture the innovation awareness of employees who are pragmatic and realistic.

Second, to maintain high productivity and innovation, organizations must ensure that their employees are focused and fully committed to their tasks (Lai et al., 2020). Therefore, organizations should recognize the importance of employee work engagement. Organizations should establish open lines of communication and encourage employees to suggest improvements and innovative ideas. Additionally, leaders should publicly praise employees’ efforts and achievements in a timely manner, as research shows that timely recognition enhances employees’ sense of accomplishment and motivation. To further support employees, organizations should respect individual needs and values. For instance, HR departments can offer flexible work arrangements (e.g., remote work options) and create personalized development plans to align with employees’ career growth aspirations. Beyond material incentives such as bonuses and pay raises, managers should also leverage spiritual incentives, such as public recognition, awards, and honors (e.g., employee-of-the-month programs), to enhance employee engagement and foster a sense of belonging.

Third, radical innovations can significantly enhance product performance and transform a company’s market position. These innovations can also fundamentally alter its technological trajectory and organizational capabilities, which are crucial for companies to achieve and maintain a sustained competitive advantage (Slater et al., 2014). Organizations should attract and retain innovative talent by implementing high-performance work systems and competitive incentive programs. Additionally, managers should provide employees with training in innovation methods and tools, such as Design Thinking, to equip them with the skills needed to drive creative solutions. To further support innovation, organizations should allocate necessary resources, such as experimental equipment and R&D funding, to enable employees to test and implement their ideas. Leaders should also foster a culture of open communication by organizing regular “Innovation Workshops” or “Brainstorming Sessions,” where employees are encouraged to freely express their ideas and collaborate on creative projects.

Fourth, conscientious leaders are goal-driven and dedicated to managing the work environment. They organize work responsibly, provide employees with necessary work resources, and structure teamwork effectively (Horowitz et al., 2006). Organizations should ensure employees have clear access to the resources they need, such as tools, equipment, information, and funds, to effectively accomplish their work. Managers can create a supportive work environment by establishing open communication channels and teamwork mechanisms. For example, regular team-building activities and cross-departmental collaboration projects can foster innovation and collaboration. Leaders should set an example by demonstrating a high level of responsibility and passion for their work. This can be achieved through public commitments and visible actions, such as actively participating in projects or recognizing team achievements. Additionally, leaders should proactively understand employees’ needs and challenges. For instance, they can hold regular one-on-one meetings or team check-ins to provide tailored support and address any obstacles.

Finally, the significance of a promotional focus has been confirmed. Employees with a promotional focus center on their ambitions, maintain enthusiasm, foster an exploratory processing style, and possess the drive to acquire knowledge and overcome challenges successfully. This forms a key foundation for creative behavior (Zhou et al., 2012). As a result, organizations should focus on fostering employees’ promotional focus. Organizations should develop facilitation-focused training programs to cultivate positive work attitudes and exploratory thinking among employees. For example, HR departments can design courses such as “Goal Orientation and Creative Thinking” to teach employees how to set aspirational goals and take proactive steps toward achieving them. During the hiring process, managers should incorporate a facilitative focus as a key assessment criterion. This can be achieved through behavioral interviews or psychometric assessments (e.g., the Big Five Personality Traits framework) to evaluate candidates’ goal orientation and exploratory tendencies. In promotion evaluations, organizations should prioritize employees who demonstrate a facilitative focus. For instance, criteria such as “innovative contribution” and “ability to achieve goals” can be used as key indicators for advancement. To further motivate employees, organizations should establish achievement-oriented incentives, such as “Innovation Achievement Awards” or “Goal Achievement Awards,” to recognize and reward those who excel in innovation and goal attainment.

### 5.3 Limitations and directions for future research

Examining leadership traits, this study provides valuable insights for enhancing employees’ radical innovations. However, there are several limitations.

First, the concept of perfectionism has evolved over time, with various scholars identifying different dimensions of it, such as normal and neurotic perfectionism (Hamachek, 1978), and perfectionist striving and concerns (Stoeber and Otto, 2006). However, this study only validated the trait of leader perfectionism based on a single dimension of other-oriented perfectionism, developed by Hewitt and Flett (1991). Therefore, future research should focus on the dimensions of a leader’s perfectionism as precursors to examine their effect on radical innovation. Additionally, these leadership styles should be compared to identify the most critical elements that affect radical innovation.

Second, this research explored the mediating role of employee work engagement in the process through which leader perfectionism predicts employee radical innovation. We discovered that in the studies conducted by Yu et al. (2021) and Park and Shin (2022), job crafting and cognitive flexibility were investigated as mediating factors. Therefore, future studies should consider examining other potential mediating variables.

Third, research on regulatory focus theory (Higgins, 1998) differentiates between promotion focus and prevention focus. This study examined only the moderating effect of promotion focus on the effect of work engagement on radical innovation, without considering the moderating effect of prevention focus. Future studies could incorporate the prevention focus variable to examine whether it negatively predicts radical innovation.

A major limitation of this study is its correlational design (correlation). Although we found a significant positive correlation between leadership perfectionism and employee engagement and radical innovation, this correlation does not prove causality. Future research could use an experimental design or a longitudinal research approach to further validate the causal relationship between variables.

Lastly, the data for this study were gathered from a single sample during the same timeframe, yielding highly similar findings, which suggests potential issues with common method bias. The first factor analysis in this study accounted for over 50% of the total variance, indicating a possible common method bias problem (Podsakoff and Organ, 1986). Consequently, future research should aim to segregate the respondents. Questions pertaining to leaders should be directed at members, while inquiries about member behavior or attitudes should be posed to leaders.

## 6 Conclusion

This study addresses a gap in understanding how a leader’s perfectionism predicts radical employee innovation within Chinese organizations, thereby expanding research on the positive effect of leadership on organizations. It also uncovers the mediating role of work engagement, exploring the connection between a leader’s perfectionism and employee work engagement, and examining radical innovation as a result of work engagement. This provides evidence that work engagement mediates the pathway through which a leader’s perfectionism predicts radical innovation. Furthermore, the study confirms the moderating effect of a leader’s conscientiousness, showing that the higher the level of conscientiousness, the stronger the effect of a leader’s perfectionism on work engagement. The moderating effect of an employee’s promotion focus was also confirmed, indicating that the higher the level of promotion focus, the stronger the effect of employee work engagement on radical innovation. These findings offer a strategy for organizations to enhance employees’ radical innovation. The results of this study can improve managers’ understanding of a leader’s perfectionism and radical innovation, providing valuable insights for organizations and managers.

## Data Availability

The original contributions presented in the study are included in the article/supplementary material, further inquiries can be directed to the corresponding author/s.
